# Increasing the provision of preventive care by community healthcare services: a stepped wedge implementation trial

**DOI:** 10.1186/s13012-017-0636-2

**Published:** 2017-08-22

**Authors:** John Wiggers, Kathleen McElwaine, Megan Freund, Libby Campbell, Jenny Bowman, Paula Wye, Luke Wolfenden, Danika Tremain, Daniel Barker, Carolyn Slattery, Karen Gillham, Kate Bartlem

**Affiliations:** 1 School of Medicine and Public Health, The University of Newcastle, Callaghan, Australia; 2 Hunter New England Population Health, Wallsend, Australia; 3 School of Psychology, University of Newcastle, Callaghan, Australia; 4 Hunter Medical Research Institute, New Lambton Heights, Australia

**Keywords:** Preventive care, Practice change, Community health care, Assessment, Advice, Referral, Smoking, Alcohol, Physical inactivity, Nutrition

## Abstract

**Background:**

Although clinical guidelines recommend the provision of care to reduce client chronic disease risk behaviours, such care is provided sub-optimally by primary healthcare providers. A study was undertaken to determine the effectiveness of an intervention in increasing community-based clinician implementation of multiple elements of recommended preventive care for four risk behaviours.

**Methods:**

A three-group stepped-wedge trial was undertaken with all 56 community-based primary healthcare facilities in one health district in New South Wales, Australia. A 12-month implementation intervention was delivered sequentially in each of three geographically and administratively defined groups of facilities. The intervention consisted of six key strategies: leadership and consensus processes, enabling systems, educational meetings and training, audit and feedback, practice change support, and practice change information and resources. Client-reported receipt of three elements of preventive care: assessment; brief advice; referral for four behavioural risks: smoking, inadequate fruit and/or vegetable consumption, alcohol overconsumption, and physical inactivity, individually, and for all such risks combined were collected for 56 months (October 2009–May 2014). Segmented logistic regression models were developed to assess intervention effectiveness.

**Results:**

A total of 5369 clients participated in data collection. Significant increases were found for receipt of four of five assessment outcomes (smoking OR 1.53; fruit and/or vegetable intake OR 2.18; alcohol consumption OR 1.69; all risks combined OR 1.78) and two of five brief advice outcomes (fruit and/or vegetable intake OR 2.05 and alcohol consumption OR 2.64). No significant increases in care delivery were observed for referral for any risk behaviour, or for physical inactivity.

**Conclusions:**

The implementation intervention was effective in enhancing assessment of client risk status but less so for elements of care that could reduce client risk: provision of brief advice and referral. The intervention was ineffective in increasing care addressing physical inactivity. Further research is required to identify barriers to the provision of preventive care and the effectiveness of practice change interventions in increasing its provision.

**Trial registration:**

Australian Clinical Trials Registry ACTRN12611001284954. Registered 15 December 2011. Retrospectively registered.

## Background

The primary health risk behaviours for the most common non-communicable diseases are tobacco smoking, poor nutrition, risky alcohol use, and physical inactivity [1, 2]. Systematic review evidence [3–6] and clinical guidelines support clinician provision of care to reduce such risk behaviours [7–9]. Clinical guidelines recommend the opportunistic provision of at least three elements of preventive care: assessment of client risk status, provision of brief advice, and referral to specialist preventive care providers or follow-up to address such risks [10].

In a number of high-income countries including Australia, ambulatory community-based healthcare services are a key setting for the delivery of care to reduce client chronic disease risk behaviours [11–13]. Limited data have been reported regarding the provision by such services of multiple elements of preventive care addressing multiple risk behaviours. Most studies have reported provision of single elements of care (risk assessment or brief advice) addressing single risk behaviours, with tobacco smoking the most frequently reported [14–16]. Across such studies, the prevalence of care provision has been reported to vary between elements of care, with a greater prevalence of risk assessment and a lower prevalence of referral/follow-up. Similarly, the prevalence of care delivery has been reported to vary between risk factors, with a greater prevalence of care for smoking and a lower prevalence for nutrition [17, 18]. These findings suggest that clinician adherence to preventive care guidelines is less than optimal, resulting in their intended clinical and population health benefits not being fully realised.

Practice change theories [19–22] and reviews of clinical practice change interventions suggest that interventions that address multiple barriers to care provision may be more likely to be effective [23, 24]. Strategies reported in systematic reviews to be effective in changing clinical practices include local consensus/leadership; enabling organisational systems; educational resources, educational meetings and support for clinicians, and clinical audit and feedback [23, 24].

Few controlled trials of implementation interventions designed to increase the provision of multiple elements of preventive care addressing multiple risks in community-based health services have been reported [25]. In a systematic review of interventions to increase the delivery of such care by primary care nurses and allied health professionals, only seven trials were identified, none of which were assessed as being of high quality [25]. Given only one trial addressed inadequate nutrition, physical inactivity, and alcohol overconsumption, no conclusions were made regarding the effectiveness of interventions in increasing care for these risks. Variable findings were reported regarding care provision for smoking.

Given the limited and equivocal evidence regarding the ability to improve community-based healthcare provision of preventive care addressing multiple chronic disease risk behaviours, a study was undertaken to assess the effectiveness of a multi-strategic implementation intervention in increasing the provision of multiple elements of preventive care for multiple risk behaviours across a network of community healthcare facilities.

## Methods

### Study design and setting

**Fig. 1:**
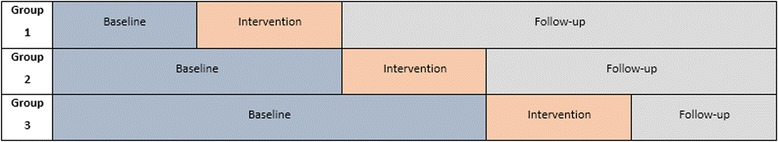
Overview of study design

A stepped-wedge trial [26, 27] was undertaken in 56 community healthcare facilities in a single health district in New South Wales, Australia [28]. The stepped-wedge design has a number of advantages for the conduct of complex and system-wide health service research; including (a) allowing for all facilities to receive and benefit from the intervention, (b) the sequential implementation of the intervention allowing for the monitoring of extraneous variables, and (c) addressing practical difficulties of recruiting a sufficient number of similar health facilities, and being more efficient as each group is used as its own control [26, 27, 29]. A 12-month implementation intervention was delivered sequentially in each of three geographically and administratively defined groups of facilities that, when combined, constituted all such facilities in the health district. Outcome data were collected for a period of 56 months for each of the three facility groups (October 2009–May 2014). The sequential delivery of the intervention resulted in baseline (control) and post-intervention follow-up periods of different length for each group (Fig. 1).The sequence of group allocation to intervention delivery was dictated by service delivery requirements. Ethical approval was obtained from the Hunter New England Human Research Ethics Committee (approval No. 09/06/17/4.03) and University of Newcastle Human Research Ethics Committee (approval No. H-2010-1116). The study protocol has been previously reported [28].

### Sample, recruitment

#### Community healthcare services

Adult community healthcare facilities that provide the following services were eligible for inclusion: community nursing, diabetes, aged care, counselling, dietetics, psychology, physiotherapy, and occupational therapist services.

Child-based services were excluded given the differential type and focus of preventive health risk behaviour care required for this client group. In-patient services were excluded based on the focus of the study being on community-based ambulatory care services. Specialist community-based services such as sexual assault, palliative care, genetics, and child protection services were excluded due the sensitive nature of such consultations.

#### Community health clinicians

The facilities employed approximately 1400 clinicians (nurses and allied health). All clinicians and managers within the eligible services were eligible to receive the intervention.

#### Clients

All adult clients who attended a face-to-face appointment were eligible to receive the intervention. A sample of approximately 20 adult clients per facility group (60 in total) was randomly selected from electronic records each week during the 56-month study period. All such clients who had at least one visit to an eligible community health service within the prior 2 weeks and who met the following inclusion criteria were eligible: 18 years of age or older, spoke English, mentally and physically capable of completing a telephone interview, not considered to be inappropriate to be contacted by a clinician, not having previously participated in the study, not involved in another community health care focused study, and not living in aged care facilities or gaol. Clients were blinded to the experimental manipulation of intervention delivery to facility groups, but not to care delivery. The selected clients were mailed an information letter and assessed for eligibility based on information from the medical record and a telephone interview.

### Model of preventive care

Clinical guidelines recommend that the opportunistic provision of preventive care occur in accordance with the “5A’s” behavioural counselling framework or variations thereof [10, 30]. Based on the 5As framework, clinicians are guided to provide care involving five elements of care: ask (systematically identify behavioural risks), advise (provide brief, tailored advice on the need to improve one or more behaviours), assess (understand willingness to change, health literacy, and agree on a plan), assist (provide behaviour change techniques, medication if appropriate), and arrange (refer to specialist behaviour change supports for ongoing support and maintenance, and/or arrange for follow-up at a later stage [10, 30].

Based on such recommendations, and taking account of competing clinical priorities and the brevity of the client consultation [10, 30, 31],clinicians were asked to provide three elements of preventive care (risk assessment for all clients, and brief advice and referral for “at risk” clients) for four risk behaviours (smoking, inadequate fruit and inadequate vegetable intake, alcohol overconsumption, and physical inactivity) in a single consultation or over successive consultations. For those clients “at-risk” according to national guidelines [1, 32], clinicians were asked to provide: brief advice; an offer of referral to free public telephone-based risk reduction services; advice to see a general practitioner/Aboriginal Medical Service, or other support service.

Prior to this study, no single model of preventive care addressing the health risks of interest was in place across community health services in the health district.

### Implementation intervention

Six types of implementation strategies, based on practice change theory and evidence demonstrating their effectiveness in modifying clinical practice [19–24] and identified barriers to the provision of preventive care by community health services, were delivered as a single package for a 12-month period in each group of services to support clinician implementation of the preventive care model. Barriers to providing preventive care were identified through extensive review of the literature and consultation with health service executives, managers, and clinician representatives. Identified barriers include perceptions of role congruence, low self-efficacy regarding ability to influence client risk behaviours, lack of leadership, competing clinical demands, insufficient training and knowledge, and limitations in information and care delivery systems [13, 31, 33, 34]. Development of the strategies was undertaken by an integrated team of behavioural and implementation scientists and population health practitioners in partnership with health service managers and relevant clinician representatives [35]. Delivery of each strategy was coordinated and led by the research team (the authors) in partnership with health service leaders and teams relevant to each strategy. Following completion of the 12-month intervention for each group, monitoring of care delivery and minimal support in response to requests by managers and clinicians continued as routine components of health district service delivery practice.

#### Leadership and consensus processes

Consensus for the model of preventive care was sought and formalised through the development of a District wide preventive care policy, that was implemented by the District Clinical Governance Office prior to the commencement of the trial (December 2010). Engagement with Health District Executives within each of the facility groups occurred prior to the intervention and was maintained during the intervention periods through monthly meetings with the research team. At the facility level, engagement by the research team with local managers and clinicians occurred prior to and throughout the intervention period. Engagement (face-to-face and telephone meetings) was undertaken prior to, and during the intervention periods to inform and to: gain the support of key stakeholders regarding the need for the intervention; to gain agreement on the need to achieve key performance targets, and to encourage advocacy for and leadership of the intervention and model of preventive care.

#### Enabling clinical and management organisational systems

Prior to intervention commencement, an existing electronic medical record system, utilised by all facilities across the district, was modified by the district information technology services. An electronic tool was developed to (1) prompt and record preventive care delivery, including standardized assessment questions for each behaviour, and for clients with one or more risks, standardised brief advice on how to improve behaviours in order to meet the Australian national guidelines, and referral to recommended referral services and/or additional local referral avenues,( 2) produce tailored client and GP/Aboriginal Medical Service information letters based on the clients’ risks and care provided, and (3) generate care delivery performance reports for managers (see audit and feedback strategy). The modifications sought to standardise previous inconsistent approaches to the prompting, focus, and recording of the elements of care for the risk behaviours of interest, through providing a single electronic template with radio button/tick box response options. At the commencement of implementation rollout in each group, a hard copy preventive care form was provided for use in home visits.

#### Clinician and manager educational meetings and training

At the commencement of the clinical practice change intervention in each group, existing clinicians were provided with online competency-based training (2 h) and 1 h of face-to-face training regarding the implementation of the model of care by the research team. Training modules covered the policy guidelines and key performance indicators; the rationale, importance and model of preventive care; the provision and recording of preventive care in the standardised electronic recording tool, service managers were provided with 2 h of face-to-face training regarding the audit and feedback strategy, and in providing leadership in preventive care. Online training modules were incorporated into existing service orientation procedures for new clinicians at the same time. Prior to this study, no single training program had been implemented across the health district that addressed the delivery of care regarding the health risks of interest.

#### Audit and feedback

Monthly care provision monitoring reports were introduced at the health district, facility and service levels. Reports included comparison against target benchmarks, and indicated the proportion of eligible clients who were assessed, and of those identified as having behavioural risks, the proportions provided with brief advice and offered referrals. Reports were emailed to and discussed with service managers by the research team on a monthly basis, commencing from the third month of the intervention in the first group of services. Where required, discussions focused on developing strategies to improve performance. Managers were encouraged to provide the reports to their staff each month.

#### Implementation support

Implementation support officers employed by the research team were allocated to each facility to provide approximately one face-to-face visit per month and fortnightly telephone support for managers, during the 12-month intervention period for each group of services. Implementation support officers provided both proactive and reactive support to managers and clinicians to facilitate clinician provision of preventive care. This may have included, for instance, supporting clinicians to use and/or trouble-shoot problems with the electronic medical records system and standardized electronic tool, disseminating resources to managers and clinicians, discussing the audit and feedback strategy with managers, and assisting service managers to develop strategies to improve performance.

#### Practice change information and resources

During face-to-face training at the commencement of the intervention for each group of services, services and clinicians were provided an email helpline, a clinician resource pack (including a process flowchart, fax-based referral forms for telephone referral services, information on Australian national guidelines for health behaviours, data entry guide, paper based assessment tools), an internet site for accessing additional clinician resources, referral information, and a workstation care provision reminder. Each month, clinicians were e-mailed tips/update information and newsletters.

### Control period

Prior to the implementation of the intervention in each group of facilities, preventive care delivery was provided according to usual practice. The level of such care has been reported to be variable and limited [36, 37]. The implementation strategies involving a preventive care policy and the modification to the electronic medical record and website, although accessible to all facilities from the initiation of the intervention for the first group of services, were operationalized and promoted according to the sequence of intervention delivery for each facility group.

### Data collection procedures

Outcome and client characteristic data were collected via client computer-assisted telephone interview conducted by trained interviewers blinded to group allocation (approximately 25 min). Additional client and service characteristics data were obtained from the clients’ electronic medical record.

### Measures

#### Client characteristics

Information collected by the telephone interview was employment status; Aboriginal and/or Torres Strait Islander status, marital status, level of education, and conditions requiring medication/medical attention. The following client information was collected from the electronic medical records: age, gender, country of birth, postcode, service type, and number of visits to the service in the prior 12 months.

#### Client risk status

Using established survey items [38–41], clients reported their risk status in the previous month for each of four risk behaviours: smoker of any tobacco products, number of serves of fruit and of vegetables usually eaten per day, frequency of alcohol consumption and number of standard drinks of alcohol consumed on a typical drinking day, how often four or more standard drinks of alcohol were consumed on a single occasion, and how many days a week they usually undertook 30 min or more of physical activity.

According to national guidelines [42–45], client risk status was defined as smoking any tobacco products [42], eating less than two serves of fruit, eating less than five serves of vegetables per day [45], drinking more than two standard drinks a day or four or more standard drinks on any one occasion [43], and engaging in less than 30 min of physical activity on at least 5 days of the week [44].

#### Client receipt of preventive care

The primary outcome measures of client-reported receipt of three elements of preventive care for each of four risk behaviours were based on items used in previous surveys [17, 36, 37, 46, 47]. Clients were asked whether a clinician asked if they smoked any tobacco products, how many serves of fruit and serves of vegetables they ate, how much alcohol they usually consumed, and how much physical activity they undertook (yes, no, do not know). For each of their risks, clients were asked whether the clinician had advised them to quit smoking or consider nicotine replacement therapy, eat more fruit or eat more vegetables, reduce their alcohol consumption, or to do more physical activity (yes, no, do not know). For each of their risks, clients were asked whether the clinician offered a referral to the Quitline for smoking, or the Get Healthy Information and Coaching service for inadequate fruit or vegetable intake and/or physical inactivity, recommended a visit to their general practitioner/Aboriginal Medical Service, or to use behaviour change support from other professional sources of support (yes, no, do not know).

### Statistical analysis

Analysis was undertaken using SAS V9.4. Client residential postcodes were used to calculate client socio-economic disadvantage [48] and geographic remoteness of residence [49].

Client reported fruit and vegetable intake was combined (fruit and/or vegetable intake), as was client reported clinician provision of care for this combined risk. For each risk behaviour, client reported receipt of each element of preventive care was dichotomised (yes/no). For each risk behaviour, receipt of referral was defined as receiving either an offer of referral to the telephone helplines, advice to use support from their General Practitioner/Aboriginal Medical Service, or advice to use support from another professional.

For the outcomes involving clinician assessment of client risk, all participants were included in the analysis. For the outcomes of clinician provision of brief advice and of referral, only those participants that reported being at-risk were included.

Analysis of receipt of care for “all risks combined” was undertaken, with such care defined as assessment for all four risks, the provision of brief advice for all of a clients’ identified risks, and the provision of any referral for all of a clients’ identified risks.

To examine change in provision of preventive care for each care element across each risk behaviour for the three groups combined, segmented logistic regression models (15 models) were used, with separate intercepts and slopes included for the three distinct time “segments”: baseline, intervention, and post intervention follow-up [50]. Clustering was controlled for by including a group term in the models. The intervention effects are expressed as odds ratios (ORs) and 95% confidence intervals (CIs).

Additional logistic regression models were applied for each group separately to provide an indication of intervention effect within groups, excluding the intervention period. Time was also included in these models so that any change in the outcome across the study period was not incorrectly attributed to the intervention. The intervention effects from these models are expressed as odds ratios (ORs) with 95% confidence intervals.

Number of visits in the last 12 months was included as a covariate in all logistic regression models. A *p* < 0.01 was used to determine statistical significance.

## Results

### Community health facilities

All 56 eligible community health facilities participated in the trial**.**

### Client characteristics

**Fig. 2:**
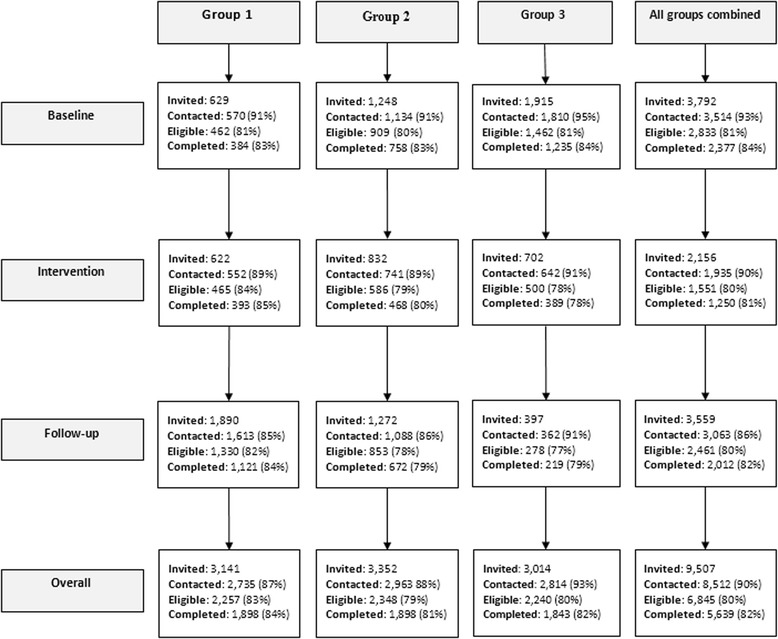
Participant eligibility and consent by cluster and time-point

Of the 9507 clients selected to participate across all three groups (3141 Group 1; 3352 Group 2; 3014 Group 3), 8512 (90%) were able to be contacted, of which 6845 (80%) were identified as eligible to participate (2257 Group 1; 2348 Group 2; 2240 Group 3). Of the eligible clients, 5639 (82%) completed data collection (1898 Group 1; 1898 Group 2; 1843 Group 3), 2377 during the “baseline” periods, 1250 during the intervention periods, and 2012 during the post intervention follow-up periods. (Fig. 2).

In the baseline and post intervention follow-up periods, 40 % of all participants were male, the majority were aged 50 or above (80%), approximately half were living with a partner (56%), and 58% were retired*.* Overall, 14% of clients were smokers, 23% consumed alcohol in excess of the Australian national guidelines, 72% did not consume recommended serves of fruit and/or vegetables, and 24% did not engage in recommended levels of physical activity. Forty-four percent of clients had 1 risk behaviour, 28% had two, 9% had three, and 1% had four. The mean number of visits to the facility in the past 12 months was 11.7 (SD 21.8) (Table 1).

### Client receipt of preventive care

**Fig. 3:**
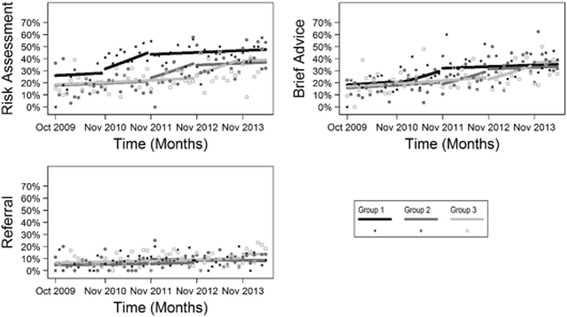
Segmented logistic regression model results. Percentage of clients reporting receiving assessment, brief advice, and referral for all risks combined

The results of the segmented logistic regressions (OR, CI’s, and *p* values) and the prevalence of each outcome for the baseline and follow-up periods are shown in Table 2. The results of these regressions are presented graphically for the three elements of care for “all risks combined” (Fig. 3).

**Table 1:** Client characteristics at baseline and follow-up for all groups

Variable	Class	Group 1		Group 2		Group 3		Overall		
		Baseline (*n* = 384; 83% consent)	Follow-up (*n* = 1121; 84% consent)	Baseline (*n* = 758; 83% consent)	Follow-up (*n* = 672; 79% consent)	Baseline (*n* = 1235; 84% consent)	Follow-up (*n* = 219; 79% consent)	Baseline (*n* = 2377; 84% consent)	Follow-up (*n* = 2012; 82% consent)	Overall (*n* = 4389; 83% consent)
Gender	Male	149 (39%)	418 (37%)	304 (40%)	248 (37%)	529 (43%)	109 (50%)	982 (41%)	775 (39%)	1757 (40%)
Age	<40	43 (11%)	212 (19%)	42 (5.5%)	56 (8.3%)	136 (11%)	25 (11%)	221 (9%0	293 (15%)	514 (12%)
	40–49	36 (9.4%)	119 (11%)	59 (7.8%)	44 (6.5%)	91 (7.4%)	10 (4.6%)	186 (8%)	173 (9%)	359 (8%)
	50–59	68 (18%)	159 (14%)	85 (11%)	78 (12%)	177 (14%)	30 (14%)	330 (14%)	267 (13%)	597 (14%)
	60+	237 (62%)	631 (56%)	572 (75%)	494 (74%)	831 (67%)	154 (70%)	1640 (69%)	1279 (64%)	2919 (67%)
Index of disadvantage^a^	Lower half	372 (97%)	1079 (96%)	664 (88%)	608 (90%)	594 (48%)	98 (45%)	1630 (69%)	1785 (89%)	3415 (78%)
	Higher half	12 (3.1%)	40 (3.6%)	94 (12%)	64 (9.5%)	641 (52%)	121 (55%)	747 (31%)	225 (11%)	972 (22%)
Client remoteness^b^	Major cities	3 (0.8%)	3 (0.3%)	1 (0.1%)	4 (0.6%)	801 (65%)	138 (63%)	805 (34%)	145 (7%)	950 (22%)
	Regional/remote	381 (99%)	1116 (99%)	757 (99%)	668 (99%)	434 (35%)	81 (37%)	1572 (66%)	1865 (93%)	3437 (78%)
Indigenous status	Aboriginal/Torres Strait Islander	17 (4.4%)	103 (9.2%)	30 (4.0%)	46 (6.8%)	34 (2.8%)	11 (5.0%)	93 (4%)	180 (9%)	273 (6%)
Marital status	Not living with a partner	177 (46%)	430 (39%)	352 (46%)	317 (47%)	576 (47%)	95 (43%)	1105 (47%)	842 (42%)	1947 (44%)
	Living with partner	206 (54%)	686 (61%)	406 (54%)	354 (53%)	657 (53%)	124 (57%)	1269 (53%)	1164 (58%)	2433 (56%)
Education level	Some high school or less	123 (32%)	272 (24%)	266 (35%)	236 (35%)	336 (27%)	63 (29%)	725 (31%)	571 (28%)	1296 (30%)
	Completed high school	195 (51%)	473 (42%)	345 (46%)	269 (40%)	554 (45%)	82 (37%)	1094 (46%)	824 (41%)	1918 (44%)
	Vocational education and training: -certificate or diploma	42 (11%)	260 (23%)	87 (12%)	107 (16%)	227 (18%)	54 (25%)	356 (15%)	421 (21%)	777 (18%)
	University: degree or higher	23 (6.0%)	114 (10%)	57 (7.5%)	60 (8.9%)	115 (9.3%)	20 (9.1%)	195 (8%)	194 (10%)	389 (9%)
Employment status	Employed	82 (21%)	342 (31%)	125 (16%)	112 (17%)	193 (16%)	30 (14%)	400 (17%)	484 (24%)	884 (20%)
	Not working	65 (17%)	115 (10%)	74 (9.8%)	88 (13%)	193 (16%)	33 (15%)	332 (14%)	236 (12%)	568 (13%)
	Other	34 (8.9%)	144 (13%)	58 (7.7%)	48 (7.1%)	97 (7.9%)	19 (8.7%)	189 (8%)	211 (10%)	400 (9%)
	Retired	203 (53%)	520 (46%)	501 (66%)	424 (63%)	751 (61%)	137 (63%)	1455 (61%)	1081 (54%)	2536 (58%)
Smoking	At risk	58 (15%)	180 (16%)	80 (11%)	98 (15%)	175 (14%)	29 (13%)	313 (13%)	307 (15%)	620 (14%)
Alcohol overconsumption	At risk	96 (25%)	292 (26%)	166 (22%)	135 (20%)	277 (22%)	46 (21%)	539 (23%)	473 (24%)	1012 (23%)
Fruit and/or vegetable intake	At risk	314 (82%)	740 (66%)	567 (75%)	428 (64%)	959 (78%)	155 (71%)	1840 (77%)	1323 (66%)	3163 (72%)
Physical inactivity	At risk	100 (26%)	275 (25%)	166 (22%)	140 (21%)	347 (28%)	44 (20%)	613 (26%)	459 (23%)	1072 (24%)
Number of risks	0	37 (9.7%)	218 (19%)	126 (17%)	164 (24%)	171 (14%)	37 (17%)	334 (14%)	419 (21%)	753 (17%)
	1	176 (46%)	451 (40%)	362 (48%)	282 (42%)	541 (44%)	109 (50%)	1079 (45%)	842 (42%)	1921 (44%)
	2	124 (32%)	335 (30%)	201 (27%)	165 (25%)	365 (30%)	57 (26%)	690 (29%)	557 (28%)	1247 (28%)
	3	40 (10%)	102 (9.1%)	61 (8.0%)	55 (8.2%)	141 (11%)	13 (5.9%)	242 (10%)	170 (8%)	412 (9%)
	4+	6 (1.6%)	15 (1.3%)	8 (1.1%)	6 (0.9%)	16 (1.3%)	3 (1.4%)	30 (1%)	24 (1%)	54 (1%)
Number of appointments in last 12 months^a^: M(SD)		14.7 (30.0)	5 (9.3)	16.6 (28.1)	13.2 (23.7)	12.6 (20.1)	13.4 (20.3)	14.2 (24.7)	8.7 (17.2)	11.7 (21.8)

^a^Socio-Economic Indexes for Areas (SEIFA) [48]. The SEIFA index of disadvantage ranks areas in Australia according to relative socio-economic advantage and disadvantage. Residential postcode was used to calculate SEIFA based on the geographic area of participant residence: lower NSW half (<=991); higher NSW half (>991)

^b^Accessibility/Remoteness Index of Australia (ARIA) [49].The ARIA is an index of the accessibility of a geographical area to a service centres (defined as an urban center with a population > = 5000). ARIA was calculated for participants based on residential postcode: Major city, ARIA <=.2; Regional or remote, ARIA >.2

**Table 2:** Proportions of clients reporting receipt of preventive care at baseline and follow-up, by group and overall

Outcome	Group 1		Group 2		Group 3		Overall	
	Baseline (*n* = 384)	Follow-up (*n* = 1121)	Baseline (*n* = 758)	Follow-up (*n* = 672)	Baseline (*n* = 1235)	Follow-up (*n* = 219)	Baseline (*n* = 2377)	Follow-up (*n* = 2012)
Assessment								
Smoking	234 (61%)	829 (74%)	375 (49%)	482 (72%)	786 (64%)	164 (75%)	1395 (59%)	1475 (73%)
Fruit and vegetable	135 (35%)	633 (56%)	202 (27%)	370 (55%)	403 (33%)	101 (46%)	740 (31%)	1104 (55%)
Alcohol	199 (52%)	761 (68%)	308 (41%)	433 (64%)	668 (54%)	152 (69%)	1175 (49%)	1346 (67%)
Physical Activity	192 (50%)	697 (62%)	349 (46%)	396 (59%)	630 (51%)	141 (65%)	1171 (49%)	1234 (61%)
All risks combined	96 (25%)	484 (43%)	125 (16%)	252 (38%)	267 (22%)	80 (37%)	488 (21%)	816 (41%)
Brief advice^a^								
Smoking	43 (74%)	142 (79%)	37 (46%)	84 (86%)	109 (62%)	23 (79%)	189 (60%)	249 (81%)
Fruit and/or vegetable	79 (25%)	305 (41%)	108 (19%)	176 (41%)	246 (26%)	56 (36%)	433 (24%)	537 (41%)
Alcohol	18 (19%)	96 (33%)	28 (17%)	53 (39%)	95 (34%)	18 (39%)	141 (26%)	167 (35%)
Physical Activity	40 (40%)	142 (52%)	66 (40%)	76 (54%)	168 (48%)	30 (68%)	274 (45%)	248 (54%)
All applicable risks combined	71 (21%)	299 (33%)	102 (16%)	185 (36%)	236 (22%)	59 (32%)	409 (20%)	543 (34%)
Referral^a^								
Smoking	7 (12%)	49 (27%)	92 (11%)	30 (31%)	25 (14%)	5 (17%)	41 (13%)	84 (27%)
Fruit and/or vegetable	36 (11%)	118 (16%)	56 (10%)	64 (15%)	134 (14%)	37 (24%)	226 (12%)	219 (17%)
Alcohol	3 (3%)	16 (5%)	6 (4%)	8 (6%)	14 (5%))	2 (4%)	23 (4%)	26 (6%)
Physical Activity	10 (10%)	52 (19%)	26 (16%)	26 (19%)	59 (17%)	13 (30%)	95 (16%)	91 (20%)
Referral for all relevant risks	19 (5%)	78 (9%)	34 (5%)	50 (10%)	95 (9%)	28 (15%)	148 (7%)	156 (10%)

^a^Limited to those who were at risk for relevant behaviour(s)

Based on the results of the segmented logistic regressions, there were significant increases in client-reported receipt of four of the five assessment outcomes: smoking (OR 1.53, 95% CI 1.14–2.06; *p* < 0.005), fruit and vegetable intake (OR 2.18, 95% CI 1.62–2.94; *p* < 0.001), alcohol consumption (OR 1.69, 95% CI 1.27–2.26; *p* < 0.001), and all risks combined (OR 1.78, 95% CI 1.28–2.47; *p* < 0.001). There were significantly increased odds of clients reporting receipt of three of the five advice outcomes: inadequate fruit and/or vegetable intake (OR 2.05, 95% CI 1.41–2.97; *p* < 0.001), alcohol overconsumption (OR 2.64, 95% CI 1.36–5.11; *p* < 0.004) and all relevant risks combined (OR 1.54, 95% CI 1.07–2.22; *p* < 0.02). No significant increases were observed for referral for any risk behaviour, individually or combined, or for the risk behaviour physical inactivity (Table 3).

**Table 3:** Estimate of the intervention effects (segmented logistic regression models)

Outcome	Intervention effect odds ratio	95% confidence interval	*p* value
Assessment			
Smoking	1.53	(1.14, 2.06)	<0.01
Fruit and vegetable	2.18	(1.62, 2.94)	<0.001
Alcohol	1.69	(1.27, 2.26)	<0.001
Physical Activity	1.12	(0.84, 1.49)	0.43
All risks combined	1.78	(1.28, 2.47)	<0.01
Advice^a^			
Smoking	1.92	(0.84, 4.41)	0.12
Fruit and/or vegetable	2.05	(1.41, 2.97)	<0.01
Alcohol	2.64	(1.36, 5.11)	<0.01
Physical Activity	1.45	(0.83, 2.53)	0.20
All applicable risks combined	1.54	(1.07, 2.22)	0.02
Referral^a^			
Smoking	2.04	(0.73, .70)	0.18
Fruit and/or vegetable	1.16	(0.72, 1.87)	0.55
Alcohol	1.38	(0.34, 5.67)	0.65
Physical Activity	1.11	(0.53, 2.32)	0.77
All relevant risks	1.41	(0.81, 2.48)	0.23

Intervention effects adjusted for group, time and number of visits to the service in the last 12 months

^a^Limited to those who were at risk for relevant behaviour(s):

Smoking risk: *n* = 313 (baseline); *n* = 307 (follow-up);Fruit and Vegetable risk: *n* = 1840 (baseline); *n* = 1323 (follow-up); Alcohol risk: *n* = 539 (baseline); *n* = 473 (follow-up); Physical inactivity risk: *n* = 613 (baseline); *n* = 459 (follow-up)

Analysis of intervention effect by group found a significant increase in care provision for fruit and vegetable assessment for Group 1 (OR 2.05, 95% CI 1.23 to 3.40; *p* < 0.006), and for Group 2 an increase in smokers receiving brief advice (OR 46.42, 95% CI 6.45 to 333.89; *p* < 0.001) (Table 4).

**Table 4:** Estimates of intervention effect by group

Outcome	Group 1		Group 2		Group 3	
	Int. effect OR	*p* value	Int. effect OR	*p* value	Int. effect OR	*p* value
Assessment						
Smoking	0.78 (0.45, 1.36)	0.38	1.61 (0.88, 2.94)	0.12	1.58 (0.96, 2.59)	0.07
Fruit and vegetable	2.05 (1.23, 3.4)	0.006	2.11 (1.13, 3.95)	0.02	1.55 (0.96, 2.5)	0.07
Alcohol	0.97 (0.57, 1.64)	0.92	1.67 (0.92, 3.03)	0.09	1.74 (1.09, 2.8)	0.02
Physical activity	0.91 (0.55, 1.52)	0.72	1.07 (0.6, 1.93)	0.81	1.21 (0.76, 1.93)	0.43
All risks combined	1.39 (0.83, 2.35)	0.22	1.74 (0.87, 3.51)	0.12	1.56 (0.92, 2.64)	0.10
Brief Advice^a^						
Smoking	0.2 (0.04, 0.95)	0.04	46.42 (6.45, 333.89)	<0.001	1.27 (0.32, 5.06)	0.73
Fruit and/or vegetable	2.17 (1.16, 4.05)	0.02	1.53 (0.68, 3.41)	0.30	1.53 (0.84, 2.77)	0.16
Alcohol	1.99 (0.65, 6.11)	0.23	4.14 (0.92, 18.61)	0.06	1.37 (0.49, 3.88)	0.55
Physical activity	1.94 (0.71, 5.35)	0.20	1.7 (0.49, 5.94)	0.41	1.4 (0.54, 3.62)	0.48
All applicable risks combined	1.74 (0.95, 3.2)	0.07	1.72 (0.78, 3.78)	0.18	1.07 (0.6, 1.89)	0.83
Referral^a^						
Smoking	2.91 (0.60, 14.07)	0.18	11.56 (1.22, 109.82)	0.03	0.57 (0.11, 3.01)	0.50
Fruit and/or vegetable	1.56 (0.67, 3.62)	0.30	0.98 (0.33, 2.89)	0.97	1.08 (0.53, 2.20)	0.83
Alcohol	2.55 (0.22, 29.33)	0.45	1.31 (0.06, 27.05)	0.86	0.57 (0.06, 5.63)	0.63
Physical activity	2.27 (0.58, 8.79)	0.24	0.91 (0.17, 4.82)	0.91	0.95 (0.32, 2.83)	0.93
Referral for all relevant risks	1.85 (0.67, 5.14)	0.24	2.24 (0.61, 8.18)	0.22	0.97 (0.44, 2.16)	0.95

Intervention Effects adjusted for time and number of visits to the service in the last 12 months

^a^Limited to those who were at risk for relevant behaviour(s)

### Intervention implementation

Implementation strategies for which monitoring data were collected are listed in Table 5, together with the proportion of facilities receiving the strategies as planned. On average per month, the delivery of implementation strategies ranged from 57% of facilities (face-to-face visit monthly) to 95% (provision of resource packs).

**Table 5:** Summary of implementation strategies provided as planned

Intervention strategies	% facilities
Leadership and consensus	
Preventive Care discussed in Executive meeting (monthly)	60%
Manager and clinician support	
Face-to-face visit (monthly)	57%
Phone/email support (fortnightly)	58%
Tips and updates provided (monthly)	70%
Preventive care newsletter provided (monthly)	62%
Performance monitoring and feedback	
Performance reports provided to managers (monthly)	92%
Performance discussed with managers (monthly)	73%
Resources	
Provision of resource packs (once off)	95%

## Discussion

The implementation intervention described in this study sought to increase the provision of multiple recommended elements of preventive care to address multiple health risk behaviours of community healthcare service clients. The study found significant increases in provision of both risk assessment and brief advice for all risks combined, and for most, but not all individual risk behaviours. No intervention effect was observed for the provision of any element of care for physical inactivity or for any measure of referral. Such findings suggest that opportunistic risk assessment and the provision of brief advice by community healthcare clinicians can be enhanced, and that further research is required to identify barriers to, and strategies to enhance clinician provision of care regarding physical inactivity and referral.

The findings of the study add to the equivocal and variable findings of a recent systematic review of interventions to increase the delivery of preventive care for multiple risk behaviours by primary care nurses and allied health professionals [25]. The findings also confirm and extend those reported in an earlier interim two-group analysis of the same study that reported effect sizes of 11–25% for provision of assessment and brief advice for inadequate fruit and/or vegetable intake, physical inactivity, and alcohol overconsumption [36]. The effect sizes in both studies were larger than those reported in previous trials of interventions to increase primary care clinician provision of preventive care [4, 14–16], and of practice change interventions generally [3–5]. The larger effect sizes may be attributable to the use of a multi-strategic approach to addressing multiple reported barriers to care delivery [25, 31, 51], or to the greater duration and the intensity of intervention implementation support provided. Further research is required to identify the mechanisms and the relative contribution of such mechanisms to enhancing the effectiveness of implementation interventions generally, and those promoting the provision of preventive care specifically [52, 53].

A key feature of this study was its focus on the provision of preventive care by non-medical primary care clinicians—nurses and allied health professionals. The results suggest that the provision of some elements of preventive care addressing multiple risk behaviours by these professional groups is both feasible and able to be increased. However, the finding that the intervention was not effective in increasing referral by such clinicians is consistent with previously reported very low levels of referral to risk reduction services by these professional groups in these settings [17, 34]. In this context of limited provision in usual care delivery, the study requirement of referral to telephone helplines represented a new form of care, one which involved additional consultation tasks, knowledge, and consultation time. The possibility exists that the intervention implementation support strategies applied in this trial were insufficient to address the novelty and additional requirements of this element of care. Implementation support strategies that have been reported to be effective in increasing clinician referral of smokers to Quitlines and other smoking cessation services [54, 55] such as: full electronic referral [56]; improved referral pathways [56]; and increasing clinician knowledge of referral options [57], may represent opportunities for increasing referral to a variety of specialist chronic disease preventive care services.

The intervention similarly did not significantly increase any element of care regarding client risk due to physical inactivity. Various organizational (reimbursement, systems, resources, support) and provider (time, skills, perception of patients’ motivation) barriers have been reported to impede the effectiveness of primary care provision of care to promote client physical activity [58, 59]. The model of care included in this trial addressed some of these barriers through a focus on just three elements of care, an emphasis on referral to external expert providers, the modification of existing organisational technology systems, and the provision of practice change support for a 12-month period. Although aligned to recommendations [58, 59], such strategies were applied generically for all risk behaviours, and were not tailored to address barriers for specific behavioural risks, such as physical activity.

The study findings should be considered in light of a number of its design and methodological characteristics. First, the use of a stepped wedge study design enabled: accounting for the effect of temporal trends through each group acting as its own “control”; the practical difficulty of recruiting a sufficient number of like community healthcare facilities to be addressed; and all community healthcare facilities to receive the intervention, a key requirement for clinician engagement [60, 61].

Second, the intervention was implemented in a single health district, thereby potentially limiting its generalisability to other districts and jurisdictions. However, as the intervention was designed based on international guidelines and practice change evidence, its ability to be tailored and scaled to accommodate variations between jurisdictions in terms of information systems, clinical processes and jurisdictional requirements is considered to be high. Third, the use of client self-report may have contributed to an overestimate of care provision [62, 63]. Fourth, the results of the ‘by group’ analyses should be interpreted with caution due to small “within group” sample sizes contributing to large effect sizes and CI’s. Finally, not all components of the implementation intervention were delivered as intended. In many cases, the non-delivery at the planned intensive frequency (monthly) occurred either as a result of staff absence (delivery or facility staff) or in response to contextual factors, such as perceived need for delivery of the strategy in a given month. It is unknown whether the non-adherence to planned intensity of strategy delivery impacted on the outcomes of the trial.

## Conclusions

The 12-month implementation intervention was effective in enhancing primary healthcare clinician assessment of client risk status, but less so for elements of care that could reduce client risk: provision of brief advice and referral. Further research is required to identify and understand the mechanisms for enhancing the effectiveness of implementation interventions for promoting preventive care, particularly in regard to referral, and for care addressing physical inactivity.
